# Digenean species diversity in teleost fishes from the Gulf of Gabes, Tunisia (Western Mediterranean)

**DOI:** 10.1051/parasite/2012192129

**Published:** 2012-05-15

**Authors:** H. Derbel, M. Châari, L. Neifar

**Affiliations:** 1 Laboratoire de Biodiversité et Écosystèmes Aquatiques, Faculté des Sciences de Sfax, Université de Sfax BP 1171 Sfax 3000 Tunisie

**Keywords:** Digenea, biodiversity, teleost fishes, Gulf of Gabes, Tunisia, Digenea, biodiversité, poissons, téléostéens, Golfe de Gabès, Tunisie

## Abstract

This study is the first attempt to survey the diversity of fish digeneans in the Gulf of Gabes (southern coast of Tunisia). A total of 779 fishes belonging to 32 species were sampled. 53 species of Digenea belonging to 15 families were recorded. Among these species, 24 are reported for the first time from the coast of Tunisia. We report one new host record, *Lecithochirium* sp. from *Sardinella aurita*. The Hemiuridae is the dominant family. A host-parasite list is presented with the information on the prevalence, abundance and mean intensity of each species collected. The diversity of Digenea is compared with other localities in the Mediterranean Sea and the northern east of Tunisia. The Gulf of Gabes shows the lowest diversity linked to the anthropogenic activities and impact of exotic species. The use of Digenea as indicators of the state of the ecosystem is discussed.

## Abstract (fr)

Cette étude est la première contribution à la connaissance de la diversité des digènes de poissons du Golfe de Gabès (côtes sud de la Tunisie). 779 poissons appartenant à 32 espèces ont été examinés. 53 espèces de Digenea appartenant à 15 familles ont été récoltées. Parmi ces espèces, 24 sont signalées pour la première fois sur les côtes de la Tunisie. Nous avons signalé un nouvel hôte, *Lecithochirium* sp., récolté de *Sardinella aurita*. Les Hemiuridae sont la famille la plus fréquente. Une liste des parasites et de leurs hôtes est présentée, en ajoutant des informations sur la prévalence, l’abondance et l’intensité moyenne de chaque espèce récoltée. La diversité des Digenea est comparée avec celle des autres localités de la Méditerranée et les côtes nord de la Tunisie. Le Golfe de Gabès possède la diversité la moins élevée à cause de l’activité anthropique et de l’impact des espèces exotiques. L’utilisation des Digenea comme indicateurs de l’état de l’écosystème est discutée.

## Introduction

The Gulf of Gabes, located in the south eastern part of Tunisia, is considered as one of the most productive areas in the Mediterranean (Boudouresque and Meinesz, 1982). It is also the most important area for fishing in Tunisia (Jabeur *et al.*, 2000). The coexistence of various industrial and urban activities in this region disrupts the stability of the ecosystem. Trawling is the most anthropogenic activity that disrupts the growth of seagrass and its associated fauna in the Gulf of Gabes (Ben Mustapha, 1995). After habitat destruction, introduced species are the second greatest threat to the local fauna.

Because of their great diversity in terms of number of species, but also because of their number of life history strategies, there is an increasing interest in using parasites as biological or ecological indicators of their fish host life conditions. Indeed, parasite communities appear to be important drivers of biodiversity, shape host population dynamics, alter interspecific competition and influence energy flow (Marcogliese, 2005). Moreover, all these factors can be influenced by environment disturbance (Sasal et al., 2007). Thus, the study of parasite communities of fishes can be used to identify contaminated habitats (Khan and Thulin, 1991; Schludermann *et al.*, 2003) and verify the equilibrium of ecosystems (Bartoli *et al.*, 2005).

Several studies of helminths have been made in the Gulf of Gabes such as Monogenea and Cestoda (;Neifar *et al.*, 2000; Neifar *et al.*, 2001; Neifar *et al.*, 2004; Derbel *et al.*, 2007). Some studies on fish digeneans have been conducted in the North of Tunisia (Gargouri Ben Abdallah & Maamouri, 2008; Gargouri Ben Abdallah *et al.*, 2010). This is the first attempt to survey the Digenea fauna off the southern coast (Gulf of Gabes). Our study aimed to list the Digenea species found in marine fish species in the Gulf of Gabes. The results presented in our paper also showed a possible use of parasites to reflect threats to the ecosystem in this region.

## Materials and Methods

Fish were caught off the coast of the Gulf of Gabes at Skhira (34° 05’ N; 10° 01’ E), Kerkennah (34° 45’ N; 11° 17’ E), and Sidi Mansour (34° 46’ N; 10° 48’ E) by local fishermen. The specimens, coming from the coastal fishing, were identified using (Fischer *et al*. 1987) and .Whitehead et al., 1984 These fish were dissected as soon as they had died and examined for digeneans. Living parasites were partially compressed beneath slide and coverslip and examined using an optical microscope. Some parasites were slightly compressed between a slide and coverslip and fixed with 70% alcohol. Some living specimens were washed in cold saline then fixed in hot saline and preserved in 5% formalin. All fixed specimens were stained with Semichon’s acetic carmine. After dehydration using graded ethanol series, the parasites were cleared in clove oil and mounted in Canada balsam for identification.

We use the diversity index M = N/N’ (N: number of parasite species/N’: number of fish species examined).

## Results and Discussion

**Fig. 1.**
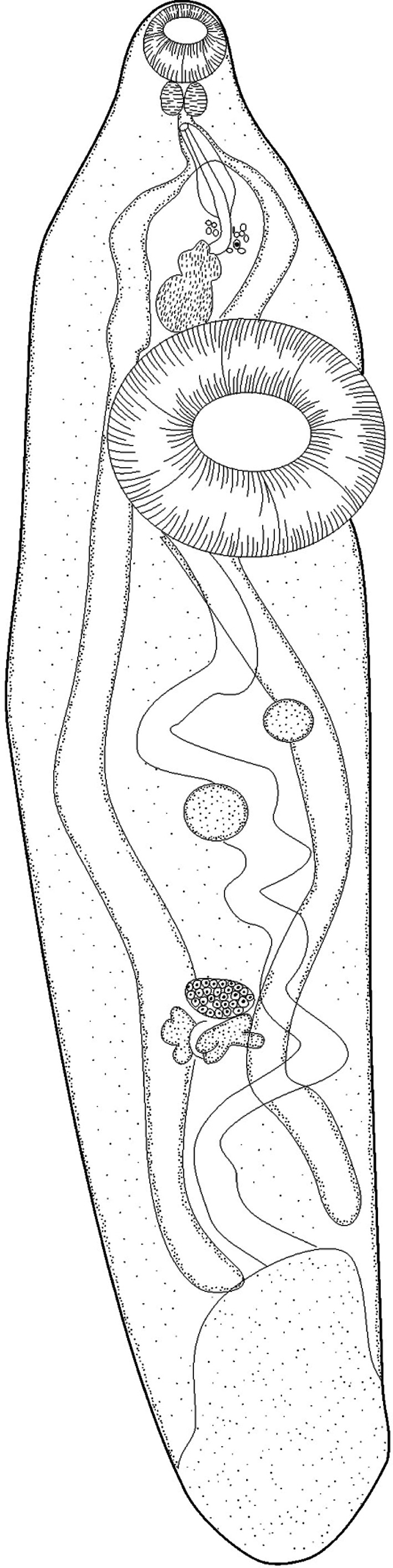
*Lecithochirium* sp. from *Sardinella aurita*. General morphology, ventral view. Scale bar = 150 μm.

During this study, 779 of teleost fishes from the Gulf of Gabes were examined for digenetic trematodes, comprising 32 species from 28 genera and 14 families. 53 species of trematodes were collected (Table I). These parasites belong to 42 genera and 15 families. 24 species, reported from Mediterranean Sea, are recorded for the first time off the coast of Tunisia (Table I). Among these species *Lecithochirium* sp. is reported from a new host *Sardinella aurita*, but it is a preadult that occurs in the swim bladder with prevalence of 13.89% *S. aurita* may be an accidental host (Fig. 1). One metacercariae, *Stephanostomum* sp. encysted on the skin of *Mullus surmuletus*.

**Table I.** List of Digenean species collected from teleost fishes of Gulf of Gabes and their epidemiologic values.

Digenea species	Digenea family	Fish species (number of specimens)	Fish family	Dates of collects (month/year)	P (%)	Abundance	Mean intensity
*Bucephalus anguillae* Špakuloá, Macko, Berrilli & Dezfuli, 2002	Bucephalidae	*Anguilla anguilla* (n = 8)	Anguillidae	12/2005	37.50	2.80	7.60
*Deropristis inflata* (Molin, 1859)	Deroprestidae	*Anguilla anguilla*	Anguillidae	12/2005	100.00	8.50	8.50
*Tergestia acanthocephala* (Stossich, 1887) *	Fellodistomidae	*Caranx crysos* (n = 16)	Carangidae	11/2005-2/2007	32.00	4.40	1.40
*Rhipidocotyle galeata* (Rudolphi, 1819) *	Bucephalidae	*Lichia amia* (n = 7)	Carangidae	8–9/2005–9/2006	100.00	33.50	33.5
*Ectenurus lepidus* Looss, 1907 *	Hemiuridae	*Trachurus trachurus* (n = 18)	Carangidae	5–10/2005–6/2005	33.00	0.33	1.00
*Monascus filiformis* (Rudolphi, 1819) *	Fellodistomidae	*Trachurus trachurus*	Carangidae	6–10/2005	33.00	0.77	2.30
*Bucephalus margaritae* (Ozaki & Ishibashi, 1934) *	Bucephalidae	*Trachinotus ovatus* (n = 5)	Carangidae	4/2005	40.00	0.50	1.50
*Lecithochirium jaffense* Fischthal, 1982 *	Hemiuridae	*Trachinotus ovatus*	Carangidae	4/2005	80.00	1.60	2.00
*Parahemiurus merus* (Linton, 1910) *	Hemiuridae	*Sardinella aurita* (n = 72)	Clupeidae	1–6/2005–7/2005	40.28	2.05	5.10
*Aphanurus stossichii* (Monticelli, 1891)	Hemiuridae	*Sardinella aurita*	Clupeidae	1–6/2005–7/2005	40.28	0.28	7.00
		*Sardina pilchardus* (n = 30)	Clupeidae	5–10/2005	76.67	1.43	1.87
		*Boops boops* (n = 18)	Sparidae	8–9–11/2005	33.33	1.38	4.10
*Lecithochirium* sp. *	Hemiuridae	*Sardinella aurita*	Clupeidae	6/2005	13.89	0.23	1.70
*Prosorhynchus aculeatus* Odhner, 1905	Bucephalidae	*Conger conger* (n = 3)	Congridae	1–6/2005	66.66	8.00	12.00
*Lecithochirium rufoviride* (Rudolphi, 1819) *	Hemiuridae	*Conger conger*	Congridae	6/2005	66.66	5.00	7.50
*Helicometra fasciata* (Rudolphi, 1819)	Opecoelidae	*Symphodus tinca* (n = 49)	Labridae	2–3/2005	49.00	4.81	9.83
		*Labrus viridis* (n = 41)	Labridae	2–3/2005	34.10	1.65	4.85
		*Sciaena umbra* (n = 14)	Sciaenidae	2–3/2005	21.40	0.35	1.66
*Schikhobalotrema sparisomae* (Manter, 1937)	Haplosplanchnidae	*Chelon labrosus* (n = 7)	Mugilidae	10/2005	28.00	2.42	8.50
		*Liza aurata* (n = 12)	Mugilidae	12/2005– 8/2007	41.66	3.08	7.40
		*Liza saliens* (n = 11)	Mugilidae	9/2006–11/2007	45.45	1.81	4.00
		*Sparisoma cretense* (n = 30)	Scaridae	9–10/2005	53.00	6.26	11.75
*Dicrogaster contracta* Looss, 1902	Haploporidae	*Liza aurata*	Mugilidae	8/2007	41.66	3.08	7.40
		*Liza saliens*	Mugilidae	11/2007	9.00	0.27	3.00
*Robinia aurata* Pankov, Webster, Blasco-Costa,	Hemiuridae	*Liza aurata*	Mugilidae	8/2007	16.66	0.33	2.00
Gibson, Littlewood & Kostadinova, 2006 *			Mugilidae				
*Saccocoelium obesum* Looss, 1902	Haploporidae	*Liza saliens*		9/2006	18.00	0.27	1.50
*Haplosplanchnus caudatus* (Srivastava, 1939)	Haplosplanchnidae	*Mugil cephalus* (n = 11)	Mugilidae	9/2006–11/2007	18.18	0.18	1.00
*Haplosplanchnus pachysomus* (Eysenhardt, 1829)	Haplosplanchnidae	*Mugil cephalus*	Mugilidae	9/2006–11/2007	63.63	1.09	1.71
*Saturnius papernai* Overstreet, 1977 *	Hemiuridae	*Mugil cephalus*	Mugilidae	11/2007	18.18	0.36	2.00
*Opecoeloides furcatus* (Lühe, 1900) *	Opecoelidae	*Mullus surmuletus* (n = 72)	Mullidae	2–3–4/2006	52.77	3.43	6.47
		*Mullus barbatus* (n = 24)	Mullidae	9/2005	45.83	4.00	8.72
*Poracanthium furcatum* Dollfus, 1948 *	Opecoelidae	*Mullus surmuletus*	Mullidae	2–3–4/2006	13.88	0.40	3.57
*Proctotrema bacilliovatum* (Odhner, 1911) *	Opecoelidae	*Mullus surmuletus*	Mullidae	2–3–4/2006	20.83	1.46	7.00
		*Mullus barbatus*	Mullidae	9/2005	25.00	1.79	7.16
*Stephanostomum* sp.	Acanthocolpidae	*Mullus surmuletus*	Mullidae	2–3–4/2006	9.72	2.91	30.00
*Prosorhynchoides arcuatus* (Linton, 1900) *	Bucephalidae	*Pomatomus saltatrix* (n = 14)	Pomatomidae	7/2005	85.00	4.57	5.30
*Paracryptogonimus aloysiae* (Stossich, 1885) *	Cryptogonimidae	*Sciaena umbra*	Sciaenidae	10/2005–7/2007	21.40	0.71	3.33
*Pleorchis polyorchis* (Stossich, 1889) *	Acanthocolpidae	*Sciaena umbra*	Sciaenidae	10/2005	7.00	0.07	1
*Lecithochirium texanum* (Chandler, 1941) *	Hemiuridae	*Euthynnus alleteratus* (n = 14)	Scombridae	11/2005–5/2007	92.85	18.14	19.53
*Lecithocladium excisum* (Rudolphi, 1819)	Hemiuridae	*Scomber japonicus* (n = 54)	Scombridae	7–8/2005	33.30	0.70	2.11
*Opechona bacillaris* (Molin, 1859) *	Lepocreadiidae	*Scomber japonicus*	Scombridae	5–7–8/2005	29.60	0.62	2.12
*Prodistomum orientalis* (Layman, 1930) *	Lepocreadiidae	*Scomber japonicus*	Scombridae	7–8/2005	7.40	0.09	1.25
*Podocotyle temensis* Fischthal & Thomas, 1970 *	Opecoelidae	*Epinephelus costae* (n = 11)	Serranidae	10/2005	27.27	4.18	15.33
*Lecithochirium musculus* (Looss, 1907) *	Hemiuridae	*Serranus scriba* (n = 21)	Serranidae	4/2005–7/2007	38.09	0.47	2.50
*Bacciger israelensis* Fischthal, 1980	Faustulidae	*Boops boops*	Sparidae	9/2005	27.77	2.00	7.20
*Robphildollfusium martinezgomezi* López-Román,	Gyliauchenidae	*Boops boops*	Sparidae	9/2005	5.55	0.16	3.00
*Gijón-Botella*, Kim & Vilca-Choque, 1992 *							
*Macvicaria crassigula* (Linton, 1910)	Opecoelidae	*Diplodus annularis* (n = 43)	Sparidae	1–4-7–11/2005	11.60	0.13	1.20
		*Diplodus vulgaris* (n = 33)	Sparidae	11–12/2005–4/2006	15.15	0.24	1.60
*Peracreadium characis* (Stossich, 1886)	Opecoelidae	*Diplodus puntazzo* (n = 19)	Sparidae	12/2005	47.36	1.10	2.33
*Diphterostomum brusinae* (Stossich, 1889)	Zoogonidae	*Diplodus vulgaris*	Sparidae	2/2005–4/2006	6.06	0.33	5.50
*Pseudopycnadena fischthali* Saad-Fares & Maillard,	Opecoelidae	*Diplodus vulgaris*	Sparidae	2/2006	6.06	0.04	1.00
1986				12/2005–2/2006			
*Aphallus tubarium* Rudolphi, 1819	Cryptogonimidae	*Dentex dentex* (n = 11)	Sparidae	2/2006	36.36	1.45	4.00
*Hemiurus communis* Odhner, 1905	Hemiuridae	*Dentex dentex*	Sparidae		18.18	0.81	4.50
*Holorchis pycnoporus* Stossich, 1901	Lepocreadiidae	*Lithognathus mormyrus* (n = 30)	Sparidae	2–5/2005	20.00	0.56	2.83
*Macvicaria mormyri* (Stossich, 1885)	Opecoelidae	*Lithognathus mormyrus*	Sparidae	2–5/2005	10.00	0.20	2.00
*Centroderma spinosissima* (Stossich, 1883)	Mesometridae	*Sarpa salpa* (n = 20)	Sparidae	1/2006–10/2007	10.00	0.95	9.50
*Lepocreadium pegorchis* (Stossich, 1901)	Lepocreadiidae	*Sarpa salpa*	Sparidae	1/2006–10/2007	30.00	3.05	10.16
*Mesometra brachycoelia* Lühe 1901	Mesometridae	*Sarpa salpa*	Sparidae	1/2006–10/2007	35.00	0.50	1.40
*Mesometra orbicularis* (Rudolphi, 1819)	Mesometridae	*Sarpa salpa*	Sparidae	1/2006–10/2007	60.00	4.20	7.00
*Robphildollfusium fractum* (Rudolphi, 1819)	Gyliauchenidae	*Sarpa salpa*	Sparidae	1/2006–10/2007	50.00	6.55	13.10
*Wardula capitellata* (Rudolphi, 1819)	Mesometridae	*Sarpa salpa*	Sparidae	1/2006–10/2007	10.00	0.10	1.00
*Allopodocotyle pedicellata* (Stossich, 1887)	Opecoelidae	*Sparus aurata* (n = 22)	Sparidae	11/2005	9.09	0.13	1.50
*Macvicaria obovata* (Molin, 1859)	Opecoelidae	*Sparus aurata*	Sparidae	11/2005	36.36	0.90	2.50
*Allopodocotyle tunisiensis* Derbel & Neifar, 2009 *	Opecoelidae	*Solea aegyptiaca* (n = 60)	Soleidae	5–9/2005– 3/2007	13.30	3.50	0.46

* First records in Tunisia.

The Hemiuridae Lühe, 1901 represents the dominant family (12 species) followed by the Opecoelidae with 11 species in the Gulf of Gabes (Table I). This result is similar to that in the North Adriatic Sea where the Hemiuridae is the predominant family (Paradižnik & RadujkoviČ, 2007). However, Opecoelidae Ozaki, 1925 is the most important family in the Scandola Nature Reserve off Corsica and off the Lebanese coast (Bartoli *et al.*, 2005;)Saad-Fares, 1985. Members of Hemiuridae generally occur in the stomach, an acid environment to which they are well adapted (Bray, 1990;)Pankov et al., 2006. The predominance of this family in the Gulf of Gabes may be a result of the resistance of this group to the environmental disturbance. (Pérez-del Olmo *et al.* 2007) showed an increase in the diversity and abundance of the hemiuroids in the post-oil spill samples off the coast of Spain. These authors related the predominance of the hemiurids to the enhancement of the populations of the benthic species such as the harpacticoid copepods, due to organic enrichment. Indeed, *Acartia* spp. are opportunistic harpacticoids which are known to serve as second intermediate hosts of a number of hemiuroids (Gibson & Bray, 1986). The analysis of the diversity of Digenea in the Gulf of Gabes shows that the most species of digeneans parasitize one host species (46 Digenea species), four were found in two host species and two were found in three host species. Some Digenea are known to be generalist in the Mediterranean Sea, such as *Diphterostomum brusinae* (Stossich, 1889), *Hemiurus communis* Odhner, 1905, and *Lepocreadium pegorchis* (Stossich, 1901). In the Gulf of Gabes, we found them in only one host fish although we examined several potential hosts. The failure transmission of digeneans to potential host may be related to environmental changes.

In this case, the helminth infects its preferential host species (Mackenzie, 1999).

The community of Digenea species shows that 16 species of fishes are parasitized by different families of Digenea species. Little interspecific competition and enough available space and resources may exist in the hosts.

In this study, there are more species of Digenea than species of fish. The number of helminth species per host species was variable. Only *Symphodus ocellatus* (n = 40), *Symphodus cinereus* (n = 36) and *Pagrus caeruleostictus* (n = 34) were entirely devoid of Digenea. By contrast, in the literature, digenean parasites are known to be present in these hosts in the Mediterranean Sea. For example, in the nature reserve off Corsica, five species were collected from *S. ocellatus* and two species from *S. cinereus* (Bartoli *et al.*, 2005). *Allopodocotyle pedicellata* (Stossich, 1887) is collected from *P. caeruleostictus* off the Lebanese coast (Saad-Fares, 1985). Among the possible reasons explaining the complete absence of certain Digenea in the Gulf of Gabes is the absence or low prevalence of the intermediate host. In addition, the environmental change can affect parasite transmission. For example, (Bartoli & Boudouresque, 1997) show the low prevalence of digenean species from *S. ocellatus* in the sites colonized by the introduced alga *Caulerpa taxifolia*. Many introduced algal species are widespread in the Gulf of Gabes such as *C. taxifolia*, *Caulerpa racemosa* and *Halophila stipulacea*. As the result of this invasion, the infralittoral communities have changed. Several authors have described the highly floristic changes, which have occurred in invaded areas with *C. taxifolia* (Verlaque & Fritayre, 1994; Villele & Verlaque, 1995). The structure of the population of most species of fish has changed, and the number of individuals and the biomass have declined significantly. As far as invertebrates are concerned, the changes are less conspicuous. It is mainly the numbers of the polychaeta and mollusc individuals which have declined. Additional sampling is necessary to support these hypotheses.

The analysis of parasite species richness of different hosts showed that *Sarpa salpa* has the richest fauna (six species). The helminth fauna of this teleost is distinct consisting mainly of members of two families (Mesometridae and Gyliauchenidae). These species have many adaptive characteristics favouring the settlement on the peculiar gut wall of this herbivorous fish and to survive in a medium rich in plant detritus. Bartoli (1987) suggested that the digeneans of *S. salpa* are not true parasites but endocommensal symbionts. So, these species are not immunogenic, or at least only slightly so, since they do not feed upon the host itself but upon its intestinal chime. In most cases this results in a high parasite density with the co-occurrence of the various species.

**Fig. 2.**
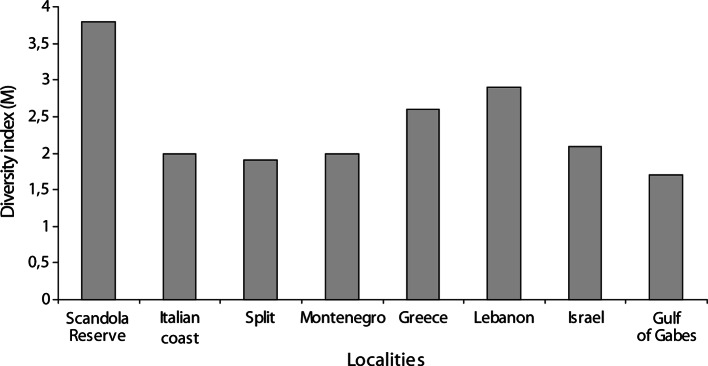
Digenean species diversity in the Gulf of Gabes and other areas of the Mediterranean. Scandola Reserve (Bartoli *et al.*, 2005), Northwestern Italian coast (Orecchia and Paggi, 1978), Split (Sey, 1970), Montenegro (Radujkovic et al., 1989), Greece (ParadIžnik and Radujkovič, 2007), Lebanon (Saad-Fares, 1980), Israel (Fischthal, 1980), Gulf of Gabes (present work).

Several authors use the diversity index M, which reflects the digenean species diversity in a specific geographical area (Bartoli *et al.*, 2005; Oguz and Bray, 2006)Keser et al., 2007. In the Gulf of Gabes this index is M = 1.7. After (Bartoli *et al.,* 2005) the highest ratio (3.8) is observed in the Scandola Nature reserve. By contrast the lowest ratio is reported for the Adriatic and North-western Italian coast (M ≤ 2), while an intermediate situation is observed for the Eastern Mediterranean (M > 2). The diversity of Digenea in the Gulf of Gabes is the lowest and closer to that found in the Adriatic (Fig. 2).

The comparison of the data reported for the Sparidae in the Gulf of Gabes with the north east of Tunisia (M = 2.9) (Gargouri Ben Abdallah & Maamouri, 2008) shows lower diversity in the Gulf of Gabes (M = 2.3). This result can be explained by the changes in the structure and the function of marine ecosystem in the south of Tunisia by human activities and the impact of exotic species. In contrast, the north coast shows lower impact of the trawling because the bottom is mainly rocky not favouring this type of fisheries (Ben Mustapha et al., 2002).

Previous studies have identified many factors influencing parasite species richness such as host traits, latitude, geographical range, phylogeny and the number of host individuals examined per species. The low diversity of Digenea in the Gulf of Gabes shows an unstable ecosystem with a decrease of the biomass and densities of hosts. In contrast, the higher digenean diversity in the Scandola Nature reserve is related to the stability of the equilibrium of the ecosystem (Bartoli *et al.*, 2005). Thus the diversity of Digenea reflects the stability of the site. Parasite communities may be good indicators of environmental disturbance because they reflect complex interactions between a possible stressor and either free-living larval stages or populations of their intermediate and final hosts (;Overstreet, 1988)Schludermann et al., 2003. On the other hand, a diverse and abundant community of parasites may be reflective of a diverse and abundant community of hosts. (Hudson *et al.* 2006) suggested that a healthy ecosystem should be one with many parasites because they reflect the presence of many different types of organisms based on the variety of complex life cycles (Marcogliese and Cone, 1997). The disturbance in the Gulf of Gabes is essentially a result of the impact of overfishing and the use of destructive fisheries such as illegal trawling causing the degradation of *Posidonia oceanica* (;Hattour, 1991 ;Ben Mustapha, 1995)Ramos-Esplá et al., 2000. A decline in the cover of *P. oceanica* has been recorded in many parts of the Mediterranean Sea, and has been attributed to several natural and anthropogenic impacts. Illegal trawling has been identified as one of the most important direct causes of large scale degradation of *P. oceanica* meadows (Martin *et al.*, 1997; Pasqualini *et al.*, 2000; González-Correa *et al.*, 2005;)Kiparissis et al., 2011. The impact of trawling on *P. oceanica* produces a reduction of canopy cover and an increase of detritus by erosion, which has an important influence on the invertebrate community (Sánchez-Jerez & Ramos-Esplá, 1996). Sea grass beds are spatially complex and biologically productive ecosystems that provide habitats and food resources for a diversified fish fauna and act as an important nursery area for many species. The damage of this ecosystem causes a qualitative and quantitative change in the structure of intermediate hosts, and therefore a modification in the frequency of Digenea fauna.

## References

[R1] Bartoli P. & Boudouresque C.F. Transmission failure of digenean parasites (Digenea) in sites colonized by the recently introduced invasive alga *Caulerpa taxifolia*. Mar Ecol Progr Series, 1997, 154, 253–260

[R2] Bartoli P., Gibson D.I. & Bray R.A. Digenean species diversity in teleost fish from a nature reserve of Corsica, France (Western Mediterranean), and a comparison with other Mediterranean regions. Journal of Natural History, 2005, 39, 47–70

[R3] Ben Mustapha K. The Gulf of Gabès: a case study in the Mediterranean decline in fishing out the Mediterranean. 21st session of the G.F.C.M Spain, Greenpeace International, Netherlands, 1995 8–9

[R4] Ben Mustapha K., Komatsu T., Hattour A., Sammari Ch., Zarrouk S., Souissi A. & El Abed A. Tunisian mega benthos from infra (*Posidonia* medows) and circalittoral (*Coralligenous*) sites. Bull Inst Natn Scien Tech Mer de Salammbô 2002, 29, 23–36

[R5] Boudouresque C.F. & Meinesz A. Découverte de l’herbier de Posidonie. Cah Parc nation Port-Cros Fr, 1982, 4, 1–79

[R6] Bray R.A. Hemiuridae (Digenea) from marine fishes of the southern Indian Ocean: Dinurinae, Elytrophallinae Glomericirrinae and Plerurinae. Systematic Parasitology, 1990, 17, 183–217

[R7] Derbel H., Boudaya L. & Neifar L. *Pseudodiplectanum syrticum* n. sp. (Monogenea: Diplectanidae), a parasite of *Synapturichthys kleinii* (Teleostei: Soleidae) from off Tunisia. Systematic Parasitology, 2007, 68, 225–231 17896190 10.1007/s11230-007-9108-4

[R8] Fischer W., Bauchot M.L. & Schneider M. Fiches FAO d’identification des espèces pour les besoins de la pêche. Méditerranée et Mer Noire. Zone de pêche 37 Vertébrés. FAO, Rome 1987, Vol. 2, 761–1530

[R9] Fischthal J.H. Some digenetic trematodes of marine fishes from Israel’s Mediterranean coast and their zoogeography, especially those from Red Sea immigrant fishes. Zool Scr, 1980, 9, 11–23

[R10] Gargouri Ben Abdallah L., Maamouri F. Digenean fauna diversity in sparid fish from Tunisian coasts. Bull Eur Ass Fish Pathol, 2008, 28 (4), 129–136

[R11] Gargouri Ben Abdallah L., Elbohli S. & Maamouri F. Digenean diversity in labrid fish from the Bay of Bizerte in Tunisia. Journal of Helminthology, 2010 84, 27–33 19580692 10.1017/S0022149X09990022

[R12] Gibson D.I. & Bray R.A. The Hemiuridae (Digenea) of fishes from the northeast Atlantic. Bulletin of the British Museum (Natural History) (Zoology), 1986, 51 (1), 1–125

[R13] González-Correa J.M., Bayle J.T., Sánxhez-Lizaso J.L., Valle C., Sánchez-Jerez P. & Ruiz J. Recovery of deep *Posidonia oceanica* meadows degraded by trawling. J Exp Mar Biol Ecol, 2005, 320, 65–76

[R14] Hattour A. Le chalutage dans les eaux tunisiennes. Réalités et considérations législatives, particulièrement dans les Golfes de Tunis et de Gabès. Notes Inst Natn Scien Tech Mer de Salammbô NS, 1991, 1, 1–26

[R15] Hudson P.J., Dobson A.P. & Lafferty K.D. Is a healthy ecosystem one that is rich in parasites? Trends in Ecology and Evolution, 2006, 21 (7), 381–385 16713014 10.1016/j.tree.2006.04.007

[R16] Jabeur Ch., Gobert B. & Missaoui H. Typologie de la flottille de pêche côtière dans le Golfe de Gabès (Tunisie). Aquat Living Resour, 2000, 13, 421–428

[R17] Keser R., Bray R.A., Oguz M.C., Çelen S., Erdoğon S., Doğuturk S., Aklanoğlu G. & Marti B. Helminth parasites of digestive tract of some teleost fish caught in the Dardanelles at Çanakkale, Turkey. Helminthologia, 2007, 44 (4), 217–221

[R18] Khan R.A. & Kiceniu J. Effects of crude oils on the gastrointestinal parasites of two species of marine fish. Journal of Wildlife Diseases, 1983, 19, 253–258 6644923 10.7589/0090-3558-19.3.253

[R19] Khan R.A. & Thulin J. Influence of pollution on parasites of aquatic animals. Advances in Parasitology, 1991, 30, 201–238 2069073 10.1016/s0065-308x(08)60309-7

[R20] Kiparissis S., Fakiris E., Papatheodorou G., Geraga M., Kornaros M., Kapareliotis A. & Ferentinos G. Illegal trawling and induced invasive algal spread as collaborative factors in a *Posidonia oceanica* meadow degradation. Biol Invasions, 2011, 13, 669–678

[R21] Mackenzie K. Parasites as pollution indicators in marine ecosystems: a proposed early warning system. Marine Pollution Bulletin, 1999, 38 (11), 955–959

[R22] Marcogliese D.J., Cone D.K. Parasite communities as indicators of ecosystem stress. Parasitologia, 1997, 39 (3), 227–232 9802071

[R23] Marcogliese D.J. Parasites of the superorganism: are they indicators of ecosystem health? International Journal for Parasitology, 2005, 35, 705–716 15925594 10.1016/j.ijpara.2005.01.015

[R24] Martin M.A., Sanchez-Lizaso J.L. & Ramos-Esplá A.A. Cuantificación del impacto de las artes de arrastre sobre la pradera de *Posidonia oceanica* (L.) Delile. Publ Espec Inst Esp Oceanogr, 1997, 23, 243–253

[R25] Neifar L., Euzet L. & Ben Hassine O.K. New species of the Monocotylidae (Monogenea) from the stingray *Dasyatis tortonesei* Capape′ (Euselachii, Dasyatidae) off the Tunisian coast, with comments on host specificity and the specific identities of Mediterranean stingrays. Systematic Parasitology 2000, 47, 43–50 10937665 10.1023/a:1006354423136

[R26] Neifar L. Euzet L. & Ben Hassine O.K. *Heteronchocotyle gymnurae* sp.n. (Monogenea: Hexbothriidae) a gill parasite of *Gymnura altavela* (Elasmobranchii: Gymnuridae) from the Mediterranean Sea. Comparative Parasitology 2001, 68, 91–96

[R27] Neifar L., Euzet L. & Oliver G. *Lamellodiscus* (Plathelminthes, Monogenea, Diplectanidae) nouveaux parasites branchiaux des poissons marins du genre *Pagrus* (Teleostei, Sparidae). Zoosystema, 2004, 26, 365–376

[R28] Oguz M.C. & Bray R.A. Digenetic trematodes of some teleost fish off the Mudanya Coast (Sea of Marmara, Bursa, Turkey). Helminthologia, 2006, 43, 161–167

[R29] Orecchia P. & Paggi L. Apetti di sistematica e di ecologia degli elminti parassiti di pesci marini studiati presso l’Istituto di Parassitologia dell’Universita di Roma. Parassitologia, 1978, 20, 73–89 553282

[R30] Overstreet R.M. Aquatic pollution problems, southeastern U.S. coasts: histopathology indicators. Aquatic *Toxicology*, 1988, 11, 213–239

[R31] Pankov P., Webster B.L., Blasco-Costa I., Gibson D.I., Littlewood D.T.J., Balbuena J.A. & Kostadinova A. *Robinia aurata* n. g., n. sp. (Digenea: Hemiuridae) from the mugilid *Liza aurata* with a molecular confirmation of its position within the Hemiuroidea. Parasitology, 2006, 133, 217–227 16623964 10.1017/S0031182006000126

[R32] Papoutsoglou S.E. Metazoan parasites of fishes from Saronicos Gulf Athens-Greece. Thalassographica, 1976, 1, 69–102

[R33] Paradižnik V. & Radujkovič B. Digenea trematodes in fish of the North Adriatic Sea. Acta Adriat, 2007, 48 (2), 115–129

[R34] Pasqualini V. & Clabaut P., Pergent G., Benyousse L. & Pergent-Martini C. Contribution of side scan sonar to the management of Mediterranean littoral ecosystems. Internat J Remote Sensing, 2000, 21 (2), 367–378

[R35] Pérez-Del Olmo A., Raga J.A., Kostadinova A. & Fernàndez M. Parasite communities in *Boops boops* after the Prestige oil-spill: detectable alterations. Marine Pollution Bulletin, 2007, 54, 266–276 17118407 10.1016/j.marpolbul.2006.10.003

[R36] Radujkovič B.M., Orecchia P. & Paggi L. Parasites des poissons marins du Montenegro : Digenes (Marine fish parasites from the Montenegro). Acta Adriat, 1989, 30 (1/2), 137–187

[R37] Ramos-Esplá A.A., Guillen J.E., Bayle J.T. & Sánchez-Jerez P. Artifical anti-trawling reefs off Alicante, South-Eastern Iberian Peninsula: evolution of reef block and set designs, *in*: Artificial Reefs in European Seas. Jensen A.C., Collins K.J., Lockwood A.P.M. (eds). Kluwer Academic publ, 2000, 195–218

[R38] Saad-Fares A. Trématodes de poissons des côtes du Liban. Spécificité. Transmission et approche populationnelle [Thesis]. Université des Sciences et Techniques du Languedoc, Montpellier 1985, 435 p.

[R39] Sánchez-Jerez P. & Ramos-Esplá A.A. Detection of environmental impacts by bottom trawling on *Posidonia oceanica* (L.). Delile meadows: sensitivity of fish and macroinvertebrate communities. J Aquat Ecosyst Health, 1996, 5, 239–253

[R40] Sasal P., Mouillot D., Fichez R., Chifflet S. & Kulbicki M. The use of fish parasites as biological indicators of anthropogenic influences in coral-reef lagoons: a case study of Apogonidae parasites in New-Caledonia. Marine Pollution Bulletin, 2007, 54, 1697–1706 17804021 10.1016/j.marpolbul.2007.06.014

[R41] Schludermann C., Konecny R., Laimgruber S., Lewis J.W., Schiemer F., Chovanec A. & Sures B. Fish macroparasites as indicators of heavy metal pollution in river sites in Austria. Parasitology, 2003, 126, S61–S69 14667173 10.1017/s0031182003003743

[R42] Sey O. Parasitic helminths occuring in Adriatic fishes. Acta Adriat, 1970, 13 (6), 1–16

[R43] Whitehead P.J.P., Bauchot M.L., Hureau J.C., Nielson J. & Tortonese E. Fishes of the north-eastern Atlantic and Mediterranean (Vol. 1). UNESCO, Paris, 1984, 510 p.

